# Correction: Lin et al. ATF3-Expressing Large-Diameter Sensory Afferents at Acute Stage as Bio-Signatures of Persistent Pain Associated with Lumbar Radiculopathy. *Cells* 2021, *10*, 992

**DOI:** 10.3390/cells13242061

**Published:** 2024-12-13

**Authors:** Jiann-Her Lin, Yu-Wen Yu, Yu-Chia Chuang, Cheng-Han Lee, Chih-Cheng Chen

**Affiliations:** 1 Division of Neurosurgery, Department of Surgery, Taipei Medical University Hospital, Taipei 110301, Taiwan; jiannher@tmu.edu.tw; 2 Department of Surgery, School of Medicine, College of Medicine, Taipei Medical University, Taipei 110301, Taiwan; yvonneyu@ibms.sinica.edu.tw; 3 Taipei Neuroscience Institute, Taipei Medical University, Taipei 110301, Taiwan; 4 Institute of Biomedical Sciences, Academia Sinica, Taipei 115201, Taiwan; ycchuang@ibms.sinica.edu.tw (Y.-C.C.); hans@ibms.sinica.edu.tw (C.-H.L.); 5 Neuroscience Program of Academia Sinica, Academia Sinica, Taipei 115201, Taiwan; 6 Taiwan Mouse Clinic, Biomedical Translation Research Center, Academia Sinica, Taipei 115202, Taiwan

## Error in Figures

In the original publication [1], there were inaccuracies in the data presentation of Figure 4. The corrected Figure 4 with the accurate data representation is provided below.

Similarly, Figure 5 contained errors in the labeling of the experimental groups. The corrected Figure 5 below ensures accurate representation of the experimental conditions and results.

## Correction to Results Sections 3.3 and 3.4

We analyze and update the results again in Results Sections 3.3 and 3.4. The corrected text for these sections is provided below.

### 3.3. The Ratios of ATF3-Positive DRG Neurons Increased Significantly After Nerve Constriction Distal to DRG Than After Nerve Constriction Proximal to DRG

Next, we tested whether the more severe DRG hypoxia due to proximal nerve constriction would result in more neuron damage compared to distal nerve constriction. Therefore, we compared the ratios of ATF3-positive DRG neurons between distal nerve constriction, proximal nerve constriction, and sham groups. In L5 DRGs, the ratio of ATF3-positive DRG neurons was significantly higher in the distal nerve constriction group than that in the proximal nerve constriction group, and the latter was significantly higher than that in the sham group at postoperative day 1 (distal vs. proximal vs. sham, 39.81 ± 2.41 vs. 23.78 ± 2.96 vs. 10.74 ± 2.17%, respectively) (Figure 4). We further analyzed ATF3 expression in different subsets of DRG neurons. In NFH-positive subpopulations, the ratio of ATF3-positive neurons in the distal constriction group was significantly higher than that in the proximal nerve constriction or sham groups, but there was no significant difference between the proximal nerve constriction and sham groups (one-way ANOVA, *p* = 0.0002, distal vs. proximal vs. sham, 57.37 ± 4.54 vs. 33.90 ± 4.80 vs. 25.55 ± 2.40%, respectively). In IB4-positive subpopulations, there was no difference among groups in the ratios of ATF3-positive neurons (one-way ANOVA, *p* = 0.0542, distal vs. proximal vs. sham, 30.91 ± 3.84 vs. 17.44 ± 3.36 vs. 17.55 ± 4.95%, respectively). In CGRP-positive subpopulations, the ratio of ATF3-positive neurons in the distal or proximal nerve constriction group was significantly higher than that in the sham group, but there was no significant difference between the distal and proximal nerve constriction groups (one-way ANOVA, *p* = 0.0225, distal vs. proximal vs. sham, 30.77 ± 3.20 vs. 32.09 ± 4.92 vs. 17.29 ± 2.53%, respectively).

### 3.4. The Ratios of CGRP-Positive DRG Neurons Increased 1 Day After Proximal Nerve Constriction

Finally, we examined the impact of different spinal nerve injuries on the expression of NFH, CGRP, and IB4-binding signals in DRG neurons 1 day after nerve constriction. The ratios of NFH-positive (one-way ANOVA, *p* = 0.8871) or IB4-positive (one-way ANOVA, *p* = 0.5993) DRG neurons were not different between groups, but the ratios of CGRP-positive DRG neurons were significantly different among the three groups (one-way ANOVA, *p* = 0.0486) (Figure 5). The ratio of CGRP-positive neurons in the proximal constriction group was significantly higher than that in the sham group (distal vs. proximal vs. sham, 29.08 ± 1.79 vs. 32.46 ± 1.55 vs. 24.95 ± 2.40%, respectively).

In addition, the information of Affiliations 1 and 2 has also been slightly adjusted. We apologize for any confusion caused and appreciate the readers’ understanding. These corrections do not affect the conclusions of the study. This correction was approved by the Academic Editor. The original publication has also been updated.

## Figures and Tables

**Figure 4:**
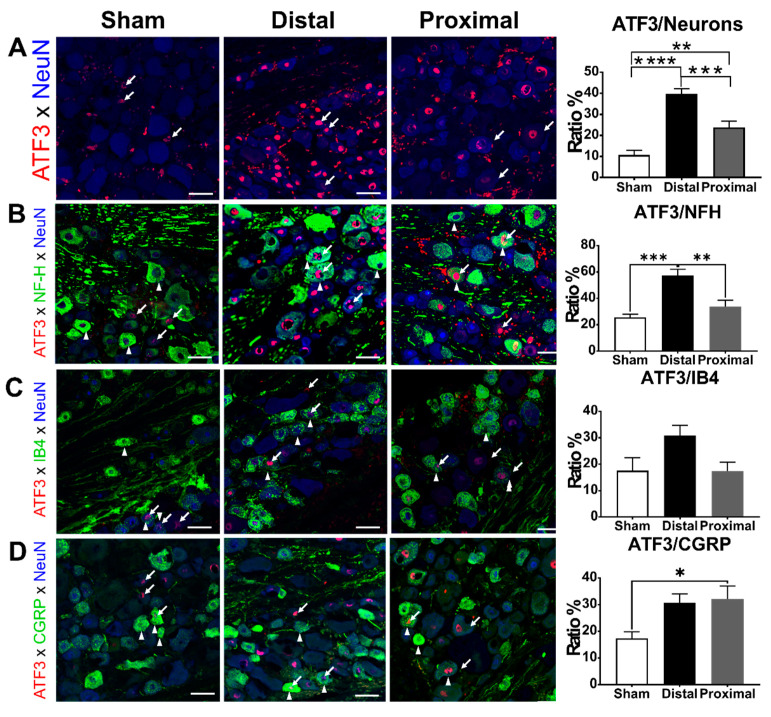
The characterization of ATF3-positive DRG neurons. The ratios of (**A**) total ATF3, (**B**) ATF3/NFH, (**C**) ATF3/IB4, or (**D**) ATF3/CGRP colocalized DRG neurons of animals with distal nerve constriction (Distal), proximal nerve constriction (Proximal), or sham operation (Sham). Data are mean ± SEM, N = 6 rats in each group. * *p* < 0.05, ** *p* < 0.01, *** *p* < 0.001, **** *p* < 0.0001, scale bar = 50 μm. Arrows indicate the ATF3 expression, arrowheads indicate a neuron subtype marker (NFH, IB4, or CGRP).

**Figure 5:**
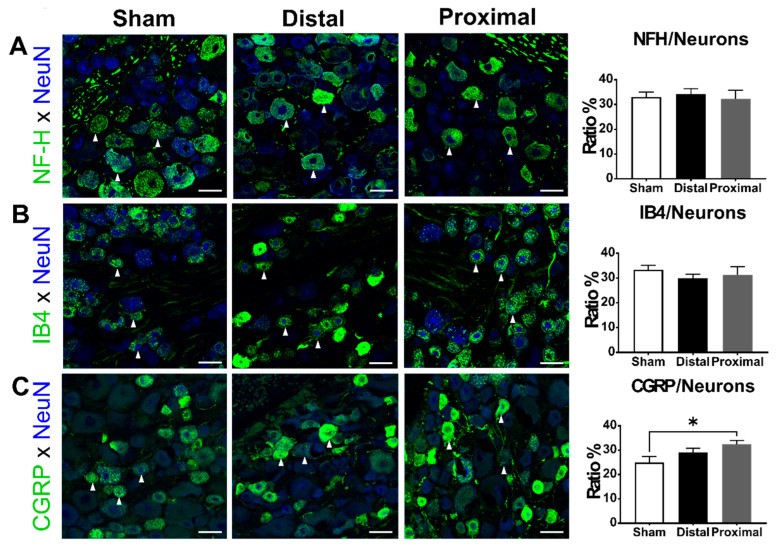
The subpopulation of DRG neurons 1 day after the operation. The ratios of (**A**) NFH, (**B**) IB4, or (**C**) CGRP-positive (arrowheads) DRG neurons of animals with distal nerve constriction (Distal), proximal nerve constriction (Proximal), or sham operation (Sham). Data are mean ± SEM, N = 6 in each group. * *p* < 0.05, scale bar = 50 μm.
